# Poorly Controlled Type 3c Diabetes due to Use of Veterinarian Insulin Syringes

**DOI:** 10.1155/2019/7916435

**Published:** 2019-06-17

**Authors:** Francisco Barrera, Natia Murvelashvili

**Affiliations:** Amita Health Saint Francis Hospital, 355 Ridge Avenue, Evanston, IL 60202, USA

## Abstract

Initiation of insulin therapy can be challenging for patients; proper training should be given by physicians and certified educators to ensure correct and safe use for outpatient population. Different insulin strengths are now available in the US; while U-40 insulin does not exist in the United States for humans, it is a very common strength used in animals. We are reporting a 30-year-old male that became insulin dependent after hospitalization for necrotizing pancreatitis who was using U-40 syringes to draw from the prescribed insulin U-100 and also presented with repeated hypoglycemic episodes.

## 1. Introduction

The proportion of patients with diabetes currently on any insulin (includes insulin only and insulin plus oral diabetes medications) was 29.1% in 2005–2012 and has been relatively stable since 1988 with 30.3% [1]. Diabetes secondary to pancreatic diseases is commonly referred to as pancreatogenic diabetes or type 3c diabetes mellitus; it is a clinically relevant condition with a prevalence of 5%-10% among all diabetic subjects in Western populations. Those with type 3c diabetes require insulin therapy more urgently than those with type 2, and most of them are discharged on insulin from the hospital [2].

## 2. Case Presentation

A 30-year-old male presented in December 2017 to the emergency department complaining of abdominal pain, which started around 3 am, and the pain was characterized as sharp, epigastric, nonradiating, and 9/10 in intensity. He admitted drinking alcohol, used to drink 5-6 glasses of wine/cocktails a day, but claimed he stopped a week ago, and he denied smoking or another illicit drug use.

Laboratory exams on admission were significant for lactic acid 3.7 (0.7-2.0 mmol/L), lipase >3000IU/L (11-82 IU/L), WBC 33,300 mmcu (4,000-11,000mmcu), and glucose 147mg/dl (70-99mg/dl). Patient underwent CT abdomen that showed acute pancreatitis. The next day patient glucose increased to 502mg/dl, bicarbonate was 18mg/dl (21-31mg/dl), and anion gap was 14. Patient then was transferred to Intensive Care Unit (ICU) for insulin infusion and management of diabetic ketoacidosis (DKA). His hemoglobin A1c was 5.5%.

Repeat CT abdomen with contrast (Figure 2) was concerning for necrotizing pancreatitis. After few days he was switched to subcutaneous insulin glargine/aspart and transferred back to general medical floor. Because patient developed fever and had persistent leukocytosis, and he was started on meropenem. His conditioned improved and antibiotics were changed to oral doxycycline and levofloxacin; he was discharged on insulin glargine 20U at bedtime and correctional insulin.

**Figure 1:**
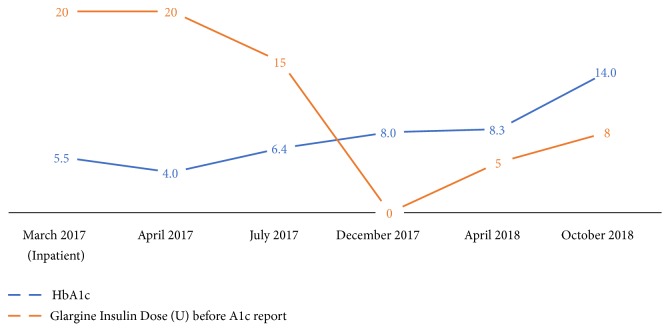
Trend of A1c and glargine insulin dose.

**Figure 2:**
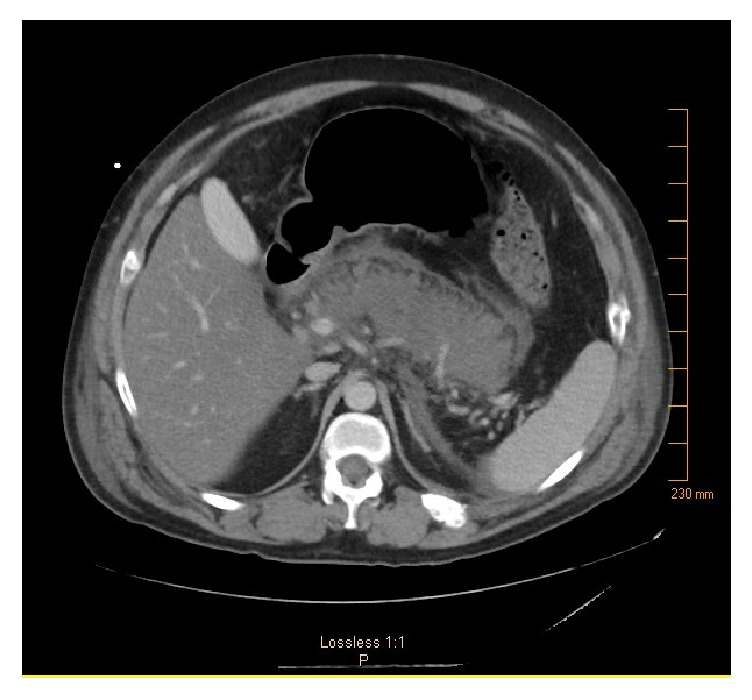
Second abdominal CT showing worsening pancreatitis.

Patient continued to follow up as an outpatient. His blood sugars were ranging between 80 and 200 and A1c at the office visit was 4% in April 2017. Glargine was decreased to 15U nightly and sliding scale with meals was continued. In June 2017 glargine was reduced by a primary care provider to 7U due to symptomatic hypoglycemia. In July 2017 at an endocrinologist's office, A1c was 6.4%, and he allegedly reported daily fasting blood sugars ranging between 70 and 200. Glargine then was stopped and patient was advised to continue taking correctional aspart insulin to administer 3U if glucose>150 (Figure 1).

In December 2017, while the patient was off glargine, A1c worsened to 8%. Patient was only using aspart as indicated and blood sugars were rarely >200s. Glargine was reinstated 5U nightly and Humalog started 4U with meals with an instruction not to take it if glucose was <110. However, in April 2018, HbA1c was 8.3% and he had episodes of symptomatic hypoglycemia. After further investigation, patient disclosed that, to avoid extra expenses, he used veterinary U-40 insulin syringes for the prescribed U100 insulin that he was getting from his wife, who herself worked in a veterinarian clinic. Unfortunately, he stopped checking his sugars regularly and on the next appointment in October 2018, HbA1c was 14%. Patient was taking glargine 8U at bedtime and Humalog 4 U if glucose was <150 and 7 U if glucose was 250-350 (Figure 1).

## 3. Discussion

There are different strengths of insulin. The standard and most commonly used strength in the United States is U-100. Insulin is labeled "U-100," which means there are 100 units of insulin per milliliter of fluid in the vial. Regular insulin is available in U-500 strength form, which is reserved for people with marked insulin resistance who take doses of more than 200 units per day [3, 4]. Concentrated insulin allows administration of smaller volumes per dose and may reduce the total number of daily injections. Until recently, regular U-500 insulin was the only concentrated insulin. Life-threatening dosing errors occurred due to lack of a U-500 syringe or availability of a U-500 insulin pen. Syringe specifically for insulin U-500 is now available; it has a green cover and 5-unit increment scale. U-500 disposable prefilled pens are also in the market [5].

New strength formulations of concentrated insulin such as glargine U-300, degludec U-200, and lispro U-200 are only available in a disposable prefilled pen; therefore, these do not carry the same safety concerns related to dosing and administration errors that U-500 insulin used to have [5, 6]. Most concentrated insulin pens give warning about not to transfer insulin from the pen into a syringe as severe overdose can happen.

Dosing errors can occur if patients are not properly educated in the use of syringes/vials and the different insulin strengths.

The syringes for administering insulin are specifically designed for each different strength. A U-100 syringe should only be used with U-100 insulin vial. Patients from North America flying overseas must bring their own insulin and syringes. In some countries the only insulin sold is in U-40 strength, syringes marked for U-40 should be used, and syringes for U-40 insulin most of the time have a red cover and red scale, rather than the orange needle cover and black scale of U-100 syringes.

Most insulin for animals is U-40 insulin and most pet owners use the specific insulin syringe U-40 to eliminate the need for dosage conversion required for a U-100 insulin syringe, which may reduce the risk of inaccurate dosing and waste [7].

1. One unit of U-100 insulin is 0.01ml in volume.
2. One unit of U-500 insulin is 0.002 ml in volume.
  (2.1) Drawing 5-unit markings of insulin U-100 vial into a U-500 syringe is 5 times less concentration than the intended dose and can result in poorly controlled diabetes [3, 4].
3. One unit of U-40 insulin is 0.025 ml.
  (3.1) Drawing 5-unit markings of insulin U-100 vial into U-40 syringe is 2.5 times greater concentration than the intended dose, resulting in hypoglycemia which is what happened with our patient.

In countries like Turkey and India, availability of both U-40 and U-100 insulin vials and syringes increases the probability of dosing error resulting in hypoglycemia or poorly controlled diabetes unless patients are informed and acquainted with both types of vials and syringes at the time of initiating insulin therapy [8, 9]. In the United Kingdom where 80% of the diabetic population are seen at special diabetes clinics, patients were switched under the direct supervision of the physician. It was the doctor's responsibility to train the diabetic patient and to issue a prescription for U-100 materials. The pharmacist collected all the U-40 materials before filling the new prescription. Conversion to U-100 insulin was postponed if the patient failed to bring old insulin and syringes to the pharmacy [8].

## 4. Conclusion

Insulin plays a very important role in the management of diabetes; however, there are many variables that can be difficult to handle for patients who are initiating its use. Diabetes education is the cornerstone of diabetes management, because diabetes requires day-to-day knowledge of nutrition, exercise, monitoring, and medication. Education and training of insulin use prior initiation of therapy can prevent multiple complications; thus, a better understanding of the different strengths of insulins and use of prefilled disposable pens will prevent rare cases like our patient who had multiple episodes of hypoglycemia and erratic diabetes management due to use of veterinarian U-40 syringes to administer U-100 insulin.
